# ADULT CHILDREN’S DIVORCE AND DEPRESSIVE SYMPTOMS IN OLDER KOREANS: THE MODERATING EFFECT OF GENDER

**DOI:** 10.1093/geroni/igae098.2724

**Published:** 2024-12-31

**Authors:** Taeeun Kim, Seolah Lee, Hyo Jung Lee

**Affiliations:** Yonsei University, Seoul, Republic of Korea; Yonsei University, Seoul, Republic of Korea; Yonsei University, Seoul, Republic of Korea

## Abstract

The study aims to investigate how adult children’s divorce is associated with older parents’ depressive symptoms in South Korea, which values Confucian ethics and collectivism. It additionally examines if parents’ gender moderates such an association. It applies the principle of linked lives, signifying that the lives of family members are closely linked to one another. Using nationally representative data from the three waves of the Korean Longitudinal Study of Aging (KLoSA) biennially conducted from 2006 to 2010, we applied mixed ANOVA, hierarchical regression analyses, and Process Macro model 1 to 1,254 parents aged 65 and over. The results indicated that parents with divorced children showed a significantly higher level of depressive symptoms over time compared to parents with only married children. In addition, children’s divorce is significantly associated with a higher level of parents’ depressive symptoms. Gender differences in depressive symptoms were observed; older mothers with divorced children reported a higher level of depressive symptoms than older mothers with only married children, whereas no significant effects were found for older fathers. The findings imply that adult children’s divorce has a negative impact on older parents’ depressive symptoms in the Korean context. Especially older mothers may suffer from stress due to their adult children’s divorce. The study also provides evidence to support the principle of linked lives through upward intergenerational transmission.

